# Membrane Anchored Immunostimulatory Oligonucleotides for In Vivo Cell Modification and Localized Immunotherapy**

**DOI:** 10.1002/anie.201101266

**Published:** 2011-06-17

**Authors:** Haipeng Liu, Brandon Kwong, Darrell J Irvine

**Affiliations:** Department of Materials Science and Engineering, Koch Institute for Integrative Cancer Research, Massachusetts Institute of TechnologyCambridge, MA 02139 (USA)Howard Hughes Medical InstituteChevy Chase, MD (USA); Department of Biological Engineering, Massachusetts Institute of TechnologyCambridge, MA 02139 (USA)

**Keywords:** cancer, cell surface modification, immunotherapy, in vivo techniques, oligonucleotides

Locally delivered immunomodulators are utilized to treat unresectable tumors and solid tumor resection sites to prevent local recurrence.^1^ Synthetic immunostimulatory oligonucleotides such as double-stranded RNA or unmethylated cytosine–guanosine motifs (CpG-ODNs) mimic molecular signatures of pathogens (viruses or bacteria, respectively) and trigger an immunostimulatory cascade including maturation, differentiation and proliferation of multiple host immune cells through pattern recognition receptors.^2^ As a result, these synthetic ODNs have been extensively studied as therapeutic agents for cancer and as vaccine adjuvants.^2^ However, a key element for the effectiveness of immunostimulatory ODNs is the close association of oligonucleotides with tumor antigen or tumor cells. For example, intratumoral/peritumoral CpG-ODN injections can lead to tumor regression in settings where intravenous CpG treatment has no effect.^3^ Also to this end, several CpG adjuvant studies indicated that co-delivery of CpG and antigens to the same antigen presenting cells (APC) significantly enhances antitumor responses.^4^ Two fundamental limitations of directly injecting ODNs into tumors are 1) relatively rapid loss of ODNs from the injection site due to their relatively low molecular weights and 2) lack of physical association between tumor cells and ODNs. We hypothesized that a membrane-interactive ODN that could spontaneously insert into cell membranes would in principle overcome both of these limitations, by prolonging ODN retention at tumor sites and more importantly, by providing a physical connection between tumor cells and ODNs.

In vitro anchoring of oligonucleotides to cell surfaces or lipid membranes has been achieved by chemical conjugation between ODNs and cell surfaces^5^ or by spontaneous insertion of lipophilic ODN conjugates into membranes (Figure 1 a).^6^ We expected the latter approach would be safer and more effective for in vivo decoration of tumor cells. To fulfill the requirements of a rapid, stable membrane anchor, we first characterized the tumor cell membrane insertion efficiency of several types of lipophilic ODNs in vitro. Fam-labeled single-stranded 20-mer oligonucleotides conjugated with cholesterol, single chain hydrocarbon (C_18_ lipid) or diacyllipids (see Figure S1 in the Supporting Information for structures) at the 5′ end were synthesized by solid phase synthesis.6b After incubating with 100 nM lipophilic ODN conjugates in PBS (phosphate-buffered saline) at 37 °C for 30 min, murine melanoma B16F10 tumor cells were washed and subsequently analyzed by flow cytometry to quantify the degree of cell surface labeling (Figure 1 b). These data revealed that cells incubated with diacyllipid ODNs had the highest fluorescence intensity (80-fold above untreated cells), while modest labeling was observed for cholesterol-ODN (48×), and single chain C_18_ lipid gave the poorest membrane insertion (3× above background, Figure 1 b). Thus under these conditions, diacyllipid tails provided the highest affinity for ODN insertion into cell membranes, consistent with previous reports where cholesterol or single alkyl tail oligonucleotide or polysaccharide conjugates have shown lower insertion levels in membranes than two-chain lipid tail conjugates.6c–e Interestingly, an attempt to minimize the charge-charge interaction between the cell surface and ODNs by inserting a poly(ethylene glycol) (PEG) spacer between the lipid tail and the oligonucleotides greatly reduced the insertion efficiency (Figure 1 b). We thus focused on diacyllipid-modified ODNs (hereafter lipo-ODNs, Figure 1 c) for our subsequent studies due to their excellent membrane affinity and low cellular toxicity.6b Confocal imaging of live cells incubated with fluorophore-labeled double- or single-stranded lipo-ODNs showed strong membrane-localized fluorescence immediately following decoration (Figure 1 d), followed by internalization of a portion of the lipo-ODNs over time at 37 °C (Figure 1 e). Variation in the concentration of lipo-ODN during cell decoration allowed the surface density of ODNs anchored on cells to be readily varied, reaching a maximum oligonucleotide density of approximately 1×10^8^ molecules per cell for 10 μM lipo-ODN (Figure S2 and Table S1).

**Figure 1:**
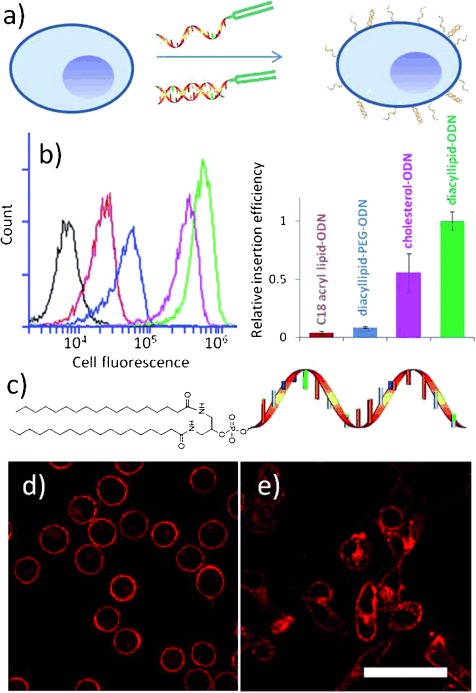
In vitro screening for optimal ODN conjugate structures. a) Schematic illustration of lipophilic-ODN insertion into cell membranes. b) Flow cytometric evaluation of membrane anchoring efficiency by different liphophilic modifications, left: flow cytometry histograms. black: untreated B16F10 cells, red: C18 single chain lipid ODN, blue: diacyllipid-PEG-ODN, purple: cholesterol-ODN and green: diacyllipid-ODN. Right: relative insertion efficiencies of each ODN conjugate based on the mean fluorescence intensity. c) Molecular structure of diacyllipid ODN. d) Confocal image of diacyllipid ODN-modified B16 cells. e) After 2 h of culture at 37 °C, a partial internalization of ODNs can be observed. Scale bar: 50 μm.

We then set up experiments to test whether in vivo cell membrane insertion would promote prolonged retention of ODNs at a tissue site. Following a common strategy to increase nuclease resistance of ODNs,^7^ we prepared a model (non-immunostimulatory) rhodamine-labeled 2′ *O*-methyl-modified RNA lipo-ODN (20-mer, see Supporting Information for sequence) for our initial in vivo studies. ODNs harboring the same sequence and fluorophore, without lipid conjugation, served as a control. C57BL/6 mice were inoculated with B16F10 cells, and when tumors reached mean sizes of 20 mm^2^, 20 μg of ODNs were injected intratumorally (Figure 2 a,b, lower injection sites) or subcutaneously into normal tissue (Figure 2 a,b, upper injection sites). After the injection, the fluorescence decay kinetics were quantitatively monitored in live animals over time using an IVIS whole animal imaging system. Since all mice were treated/imaged under identical conditions, we reasoned that a direct fluorescence comparison would be valid for evaluating local ODN pharmacokinetics, although multiple parameters, including ODN diffusion into circulation, nuclease degradation, and photo-bleaching could all contribute to the fluorescence decay with time.

**Figure 2:**
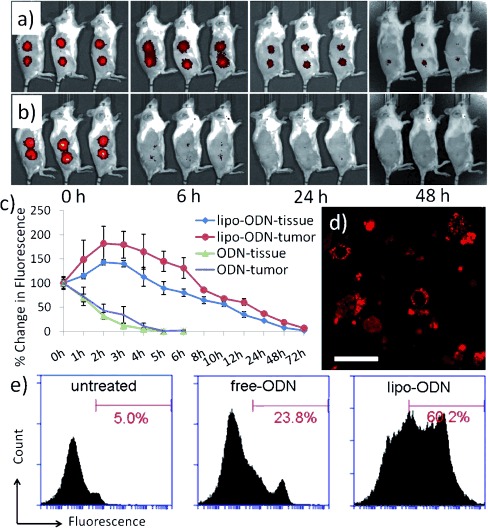
In vivo cell modification by lipo-ODNs. a,b) In vivo kinetics of fluorescence decay of rhodamine-conjugated lipo-ODN (a) and non-lipidated ODN (b). The upper sites on mice were subcutaneous injections into healthy tissue; the lower sites were intratumoral injections. c) Quantification of total fluorescence over time from IVIS whole-animal imaging of injection sites. d, e) Representative confocal image of tumor cells recovered from an intratumoral injection site (d) and flow cytometric analysis of recovered tumor cells (e) 3 h after injection. Scale bar: 50 μm.

Consistent with our hypothesis, the lipo-ODN remained at the injection site far longer than the free-ODN control, in both healthy tissue and tumor sites (Figure 2 a–c). The observed fluorescence intensities, based on the fluorescence quantification, revealed a half life (*t*_1/2_) of 10 h for lipo-ODN as compared to *t*_1/2_ of 1.5 h for the unconjugated ODN control. The fluorescence pattern of lipo-ODN showed an initial increase over the first few hours, reaching a maximum intensity 3 h after the injection, which we believe reflects unquenching of fluorophores as the micellar lipid-oligonucleotides insert into membranes. This was followed by a prolonged retention with fluorescence decaying to baseline over 72 h after injection. In contrast, the unconjugated control ODN showed a rapid decay to background over about 4 h. Decay kinetics at healthy tissue sites were similar to those following intratumoral injection, suggesting similar cell insertion/clearance mechanisms in both cases. Since the hydrophobic lipid moieties are the only difference between these two ODNs, we reasoned the rapid decrease of fluorescence at the site of injection was due to diffusion and convection of the free oligonucleotides, whereas the prolonged fluorescence by lipo-ODN was a result of local cell membrane insertion. To test this idea, we examined cells recovered from tumor injection sites. Tumors were excised 3 h after injection, a single cell suspension was prepared from the entire tumor, and the cells were immediately analyzed live by flow cytometry and confocal microscopy. Confocal microscopy analysis showed that similar to our in vitro data, tumor cells recovered from tumors injected with lipo-ODN were loaded with substantial quantities of both surface and internalized lipo-ODN (Figure 2 d, Figure S3). In tumors injected with lipo-ODN, 60 % of the of the recovered tumor cells were rhodamine^+^, while only 24 % of cells recovered from tumors treated with unmodified ODN had fluorescence above background (Figure 2 e). Thus, the prolonged retention at the injection site is at least partially accounted for by insertion in local cell membranes and internalization of the amphiphilic oligonucleotides.

To determine whether enhanced tumor cell association/retention at tumor sites could enhance the therapeutic efficacy of immunostimulatory ODNs, we next turned our attention to an unmethylated CpG single-stranded DNA oligonucleotide. CpG ODNs containing cytosine-guanosine motifs can trigger an immunomodulatory cascade that involves multiple immune cells.^2^ Previous studies suggest a close association between CpG ODN and tumor cells is required for an effective treatment.^3^ This has led to the preference of intratumoral administration and the concept of co-delivery of CpG with tumor antigens for cancer vaccines. We prepared a fluorescein labeled lipo-CpG ODN with unconjugated CpG and lipo-GpC, a non-stimulatory sequence, as controls (see Supporting Information for sequences). Previous studies reported that derivatization of CpG sequences, especially at the 5′ end, can abrogate its immunostimulatory efficacy.^8^ We thus first tested whether lipo-CpG retained its bioactivity in vitro. Bone marrow dendritic cells (DCs) were incubated with 500 nM lipo-CpG, and 12 h later secretion of the inflammatory cytokines interleukin (IL)-6 and IL-12 were assayed in the culture supernatant by ELISA. Notably, lipid-conjugated CpG stimulated similar cytokine production from DCs as unmodified CpG, while control lipo-GpC with a nonstimulatory sequence elicited no cytokine production above background (Figure S4), showing that lipid conjugation did not block the specific immunostimulatory activity of this ODN sequence.

We then injected a single intratumoral dose of dye-labeled lipo-CpG (20 μg) or soluble CpG into established B16F10 melanoma tumors and measured the fluorescence decay kinetics at the injection site by IVIS as before. Interestingly, unconjugated phosphorothioate CpG showed a much longer local retention (Figure S5, *t*_1/2_=24 h) than the 2′OMe-modified RNA oligonucleotides tested above (*t*_1/2_=1.5 h). This likely reflects a combination of greater nuclease resistance of the DNA oligonucleotides and the tendency for nonspecific binding of the phosphorothioate backbone ODNs to the tissue.^9^ Nevertheless, lipo-CpG-fam again showed prolonged fluorescence kinetics when compared with free CpG, with 15 % of CpG fluorescence still detectable at the injection site after 12 days, 10-fold greater than the quantity of free CpG detectable at this time point (1.4 %, Figure S5).

To investigate the potential therapeutic benefits of lipo-CpG, B16F10 tumors were treated by two intratumoral injections of 20 μg lipo-GpC (control), CpG, or lipo-CpG on day 4 and day 8 after inoculation of 5×10^5^ tumor cells. As shown in Figure 3, local treatment with lipo-CpG oligonucleotides inhibited tumor growth over several weeks. In contrast, treatment with unconjugated CpG inhibited tumor growth only until day 18, after which time tumors rapidly progressed with all the animals succumbing by day 33, a time when half of the lipo-CpG-treated animals were still alive (Figure 3 b). Notably, control experiments using a lipo-GpC sequence did not show any antitumor activity, suggesting that the therapeutic benefit is due to the specific immunostimulatory effects of the Toll-like receptor 9-binding CpG motif. Importantly, animals treated with lipo-CpG did not exhibit signs of significant local or systemic toxicity, changes in body weight or ambulation, suggesting a lack of toxic side effects on healthy tissue that might be exposed to the lipid conjugate. These observations demonstrate that this method of enhancing the local retention of immunostimulatory ODNs within the tumor milieu is indeed beneficial for tumor immunotherapy.

**Figure 3:**
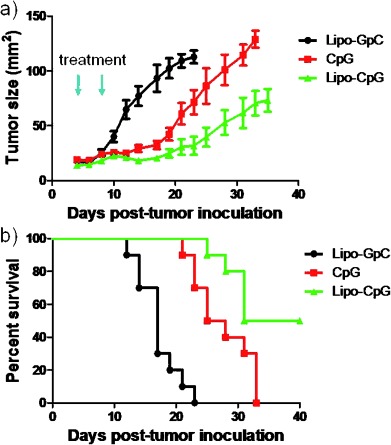
Therapeutic effects of lipid modified CpG ODN. a) Time course analysis of tumor growth (*n*=10) after treated with two injections of either lipo-GpC, CpG or lipo-CpG. The differences for the treatment of lipo-CpG versus CpG were statistically significant (*P*<0.004, paired t-test). b) Kaplan–Meier survival curve (with log-rank test) after treated with ODN probes, tumor-bearing mice treated with lipo-CpG have a prolonged survival compared with CpG group (*P*<0.01).

In summary, we have demonstrated a facile and simple method for in vivo cell modification with single-stranded or double-stranded immunostimulatory oligonucleotides. Local injection of membrane anchored ODN not only promoted an in situ membrane insertion, resulting in a higher local concentration of ODN within the tumor microenvironment over a prolonged period of time, but also promoted physical association of ODNs with tumor cells. In vivo modification of tumor cells will be beneficial for the local stimulation of antigen presenting cells such as dendritic cells responding to apoptotic/necrotic tumor cells. We also demonstrated a therapeutic benefit of this strategy by using a lipid-conjugated immunostimulatory ODN. This strategy could be immediately extended to many other functional ODNs, for example, immunostimulatory RNAs, siRNA, DNAzymes, or aptamers.

## Notes

Detailed facts of importance to specialist readers are published as ”Supporting Information”. Such documents are peer-reviewed, but not copy-edited or typeset. They are made available as submitted by the authors.
