# Supplementary material for: Membrane Anchored Immunostimulatory Oligonucleotides for In Vivo Cell Modification and Localized Immunotherapy
Source: Angew Chem Int Ed Engl. 2011 Jun 17;50(31):7052–5. doi: 10.1002/anie.201101266 (PMC3166645; doi:10.1002/anie.201101266)
Supplement: Supplementary file 1 [file anie0050-7052-SD1.pdf]

Angewandte Chemie
Eine Zeitschrift der Gesellschaft Deutscher Chemiker

Supporting Information

© Wiley-VCH 2011

69451 Weinheim, Germany

Membrane Anchored Immunostimulatory Oligonucleotides for In Vivo Cell Modification and Localized Immunotherapy**

*Haipeng Liu, Brandon Kwong, and Darrell J. Irvine**

anie_201101266_sm_miscellaneous_information.pdf

Materials and methods

Materials

Unless otherwise stated, all solvents and chemicals were obtained from Sigma-Aldrich without further purification. HPLC was performed on a Shimadzu Prominence system; UV/Vis was recorded by a ultrospec 2100 pro UV/Visible spectrophotometer (Amersham pharmacia biotech); ^{1}H NMR, ^{31}P NMR were recorded on a Varian Mercury (300MHz) spectrometer using tetramethylsilane (TMS) as internal standard, chemical shifts are reported in ppm (δ) referenced to TMS. Oligonucleotides were synthesized in 1.0 micromolar scale on an automated DNA synthesiszer (ABI 394, Applied Biosystems, Inc.). All DNA synthesis reagents including cholesteryl-TEG phosphoramadite and DMT-PEG-phosphoramadite were purchased from Glenres and Chemgenes and used by following manufacturer's instructions.

Synthesis of C18 lipid phosphoramidite:

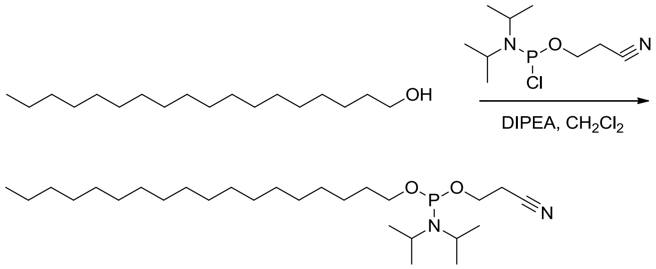

1-octadecanol (2.7 g, 10 mmol), and DIPEA (5.2 mL, 30 mmol) was dissolved in 100 mL dichloromethane, the mixture was cooled to $0^{o}C$ and 2-Cyanoethyl N,N-diisopropylchloro phosphoramidite (2.84 g, 12 mmol) was added dropwise under dry nitrogen. The reaction was allowed to warm up to room temperature for 1 hour. After dilution with EtOAc, the solution was washed with saturated $NaHCO_3$ and brine and dried over Na_2SO_4. The solution was evaporated and the residue was purified by chromatography over silica gel using 25% EtOAc:65% hexanes: 10% Et_3N to provide final product (90%) as a colorless oil. ^{1}H NMR ($CDC1_3$, 300 MHz): 0.86 (t, 3H), 1.1-1.5 (m, 30H), 1.35 (d, 12H), 1.60 (t, 2H), δ 3.5-3.7 (m, 4H), 3.7-3.9 (m, 2H). ^{31}P NMR ($CDCl_3$) 147 ppm.

Synthesis of diacyllipid phophoramidite:

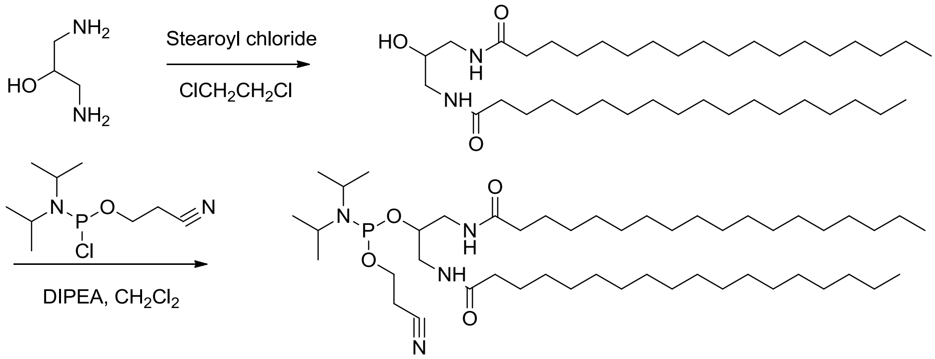

The diacyllipid phosphoramidite was synthesized in two steps as described before:[1]

A solution of stearoyl chloride (6.789 g, 22.41 mmol) in $ClCH_2CH_2Cl$ (50 ml) was added dropwise to a solution of 1,3-diamino-2-dydroxypropane (1.0 g, 11.10 mmol) in the presence of $ClCH_2CH_2Cl$ (100 ml) and triethylamine (2.896 g, 22.41 mmol). The reaction mixture was stirred for 2 hours at room

temperature and then heated at 70 ℃ overnight. The reaction mixture was then cooled to RT, filtered, and the solid was washed with CH_2Cl_2, CH_3OH, 5% $NaHCO_3$ and diethyl ether, respectively. The solid was dried under vacuum to give the intermediate product as a white solid (yield: 90%). ^{1}H NMR (300 MHz, $CDCl_3$): δ 6.3 (m, 2H), 3.8 (m, 1H), 3.4-3.2 (m, 4H), 2.2 (t, 4H), 1.6 (m, 4H), 1.3-1.2 (m, 60H), 0.9 (t, 6H). The intermediate product (5.8 g, 9.31 mmol) and DIPEA (4.2 mL, 18.62 mmol) was then dissolved in anhydrous CH_2Cl_2 (100 ml). The solution was cooled on an ice bath and 2-Cyanoethyl N,N-diisopropylchlorophosphoramidite (8.6 mL, 0.47 mmol) was added dropwise under dry nitrogen. After stirring at RT for 1 hour, the solution was heated to 60 ℃ for 90 minutes. The reaction mixture was washed with 5% $NaHCO_3$ and brine, dried over Na_2SO_4 and concentrated under vacuum. The final product was isolated by precipitation from acetone to afford 4g (55% yield) phosphoramidite as a white solid. ^{1}H NMR (300 MHz, $CDCl_3$): δ 6.4 (m, 2H), 3.9 (m, 2H), 3.8 (m, 2H), 3.6 (m, 2H), 3.0-2.9 (m, 2H), 2.6 (t, 2H), 2.2 (m, 4H), 1.6 (m, 6H), 1.3-1.2 (m, 72H), 0.9 (t, 6H). ^{31}P NMR ($CDCl_3$) 154 ppm.

DNA synthesis and lipophilic conjugation

All DNA and RNA sequences were synthesized using an ABI 394 synthesizer on 1.0 micromole scale. All lipophilic phosphoramidites were conjugated as a final 'base' on 5' end of the oligos. Lipophilic phosphoramidites were dissolved in dichloromethane and coupled to oligos by using the so-called syringe synthesis technique.[2] Briefly, lipid phosphoramidites (200 μL) were mixed with activator (0.2 mM 5-Ethylthio Tetrazole in 200 μL Acetonitrile), and the mixture were pushed back and forth through the CpG column using 2 syringes for 10 min. Alternatively, lipophilic phosphoramidite could also be coupled using the DNA synthesizer (15 min coupling time). After the synthesis, DNA was cleaved from the CpG and deprotected and purified by reverse phase HPLC using a C4 column (BioBasic-4, 200mm x 4.6mm, Thermo Scientific), 100 mM triethylamine-acetic acid buffer (TEAA, pH 7.5)-acetonitrile (0-30 min, 10-100%) as an eluent. Lipophilic ODNs typically eluted at 20 min while unconjugated ODNs eluted at 8 min. Immunostimulatory CpG oligos employed were a type B sequence known as 1826.[3]

ODN sequences:
Cholesteryl ODN: 5'-Cholesteryl- AAA AAA AAT CAC AGA TGA GT -Fluorescein-3'
C18 lipid ODN: 5'-C18 lipid-AAA AAA AAT CAC AGA TGA GT-Fluorescein-3'
Diacyllipid ODN: 5'-diacyllipid- AAA AAA AAT CAC AGA TGA GT-Fluorescein-3'
Diacyllipid PEG ODN: 5'-diacyllipid-$[(CH_2\text{-}CH_2\text{-}O)_6\text{-}PO_3]_4$-AAA AAA AAT CAC AGA TGA GT-Fluorescein-3'
Lipo-ODN: 5'-diacyllipid-cgg auu acc agg gau uuc aTT-TMR-3'
Free-ODN: 5'-cgg auu acc agg gau uuc aTT-TMR-3'
Lipo-CpG-fam: 5'diacyllipid-*T*C*C* A*T*G* A*C*G* T*T*C* C*T*G* A*C*G* T*T*-Fluorescein-3'
Lipo-GpC-fam: 5'diacyllipid-*T*C*C* A*T*G* A*G*C* T*T*C* C*T*G* A*G*C* T*T*-Fluorescein-3'
CpG-fam: 5'-T*C*C* A*T*G* A*C*G* T*T*C* C*T*G* A*C*G* T*T*- Fluorescein-3'
Lipo-CpG: 5'diacyllipid-*T*C*C* A*T*G* A*C*G* T*T*C* C*T*G* A*C*G* T*T*-3'
Lipo-GpC: 5'diacyllipid-*T*C*C* A*T*G* A*G*C* T*T*C* C*T*G* A*G*C* T*T*-3'
CpG: 5'-T*C*C* A*T*G* A*C*G* T*T*C* C*T*G* A*C*G* T*T*-3'

Note: low case letters: 2'OMe RNA, Capital letters: DNA, * phosphorothiolate backbone

Animals and cells

Animals were cared for in the USDA-inspected MIT Animal Facility under federal, state, local and NIH guidelines for animal care. C57BL/6 albino mice (6-8 weeks) were obtained from the Jackson Laboratory. Bone marrow-derived dendritic cells were prepared following a modification of the procedure of Inaba[4]

as previously reported. DCs were activated/matured with 500 nM CpG probes for 12 h and washed 3X with PBS before use. B16F10 parental melanoma cells were obtained from American Type Culture Collection. Cells were cultured in complete medium (MEM, 5% fetal bovine serum (Greiner Bio-one), 100 U/ml penicillin G sodium and 100 µg/ml streptomycin (Pen/Strep), MEM sodium pyruvate (1 mM), NaH_2CO_3, MEM vitamins, MEM non-essential amino acids (all from Invitrogen), 20 µM β-mercaptoethanol (β-ME)).

Tumor inoculation and CpG therapy

For tumor implantation, anesthetized C57/BL6 mice (groups of 10) were inoculated s.c. into the left flank with 5×10^5 B16F10 cells suspended in 50 µl of PBS, which were then allowed to establish for 4 days (at this time, tumors were clearly visible with mean sizes of ~18 mm^2). Mice were treated on days 4 and day 8 after tumor inoculation with intratumoral injections of 20 µg CpG in soluble or lipid-conjugate form (50 µL in PBS). Tumor growth was followed by measuring two orthogonal diameters and tumor size was calculated by using the formula: Tumor size = Length * Width.

Tumor digestion and confocal microscopy

Tumors were excised 3 hours after probes injection and a single cell suspension was prepared from the entire tumor by mincing the tissue and filtering the suspension through 30 µm nylon mesh. Tumor cells were then washed 3 times with PBS and samples were studied by laser scanning confocal microscopy (Zeiss LSM 510).

Quantitation of Cell Surface Oligonucleotides:

To avoid the problematic pH-sensitive nature of Fluorescein (Fam), we chose a rhodamine dye for quantitation measurements. Briefly, $1x10^6$ B16F10 cells were suspended in PBS and various diacyllipid ODN-TMR (sequence: 5'-diacyllipid- AAA AAA AAT CAC AGA TGA GT-TMR-3') were mixed and incubated at 37°C for 20 min. After washing three times with PBS the cells were lysed by Glo lysis buffer (Promega) and fluorescence was quantified by a plate reader. After subtracting cellular autofluorescence the readouts were fit to a standard curve to calculate the ODN concentrations. Cell diameter was estimated to be 20 µm.

Table S1 Quantitation of cell surface oligonucleotides

ODN concentration	ODNs(pg)/cell*	Number of ODNs/cell	Coverage**
10 µM	1.159474+/-0.046 pg	$1.05758x10^8$	15.5%
5 µM	0.858924+/- 0.025 pg	$7.83443x10^7$	9.79%
2 µM	0.647850+/-0.022 pg	$5.90583x10^7$	7.38%
1 µM	0.507831+/-0.054 pg	$5.153597x10^7$	6.44%
500nM	0.443046+/-0.033 pg	$4.497395x10^7$	5.62%
200nM	0.326015+/-0.043 pg	$3.305025x10^7$	4.13%
100nM	0.254960+/-0.027 pg	$2.584802x10^7$	3.23%
50nM	0.200624+/-0.044 pg	$2.032630x10^7$	2.54%

*see curve fitting in Figure S2.

** % coverage was estimated based on 20 µm cell diameter and 2 nm diameter of each ODN.

Statistical analysis:

All values are reported as means ± SE. The two-tailed paired t-tests was used to compare the difference in tumor growth. Survival curves were created by the Kaplan-Meier method and compared by a log-rank test. A value of $P < 0.05$ was considered statistically significant. All statistical analyses were performed using GraphPad Prism 5.0 (GraphPad Software, Inc. La Jolla, CA).

Supplemental References:

1. H. Liu, Z. Zhu, H. Kang, Y. Wu, K. Sefan, W. Tan, *Chem. Eur. J.*, **2010**, *16*, 3791-3194.
2. J. J. Storhoff, R. Elghanian, R. C. Mucic, C. A. Mirkin, R. L. Letsinger, *J Am. Chem. Soc.* **1999**, *120*, 1959-1964.
3. A. K. Ballas, A. M. Krieg, T. Warren, W. Rasmussen, H. L. Davis, M. W. Waldschmidt, G. Weine, *J. Immunol*. **2001**, *167*, 4878-4886.
4. K. Inaba, M. Inaba, N. Romani, M. Aya, M. Deguchi, S. Ikehara, *J. Exp. Med.* **1992,** *176,* 1693–1702.

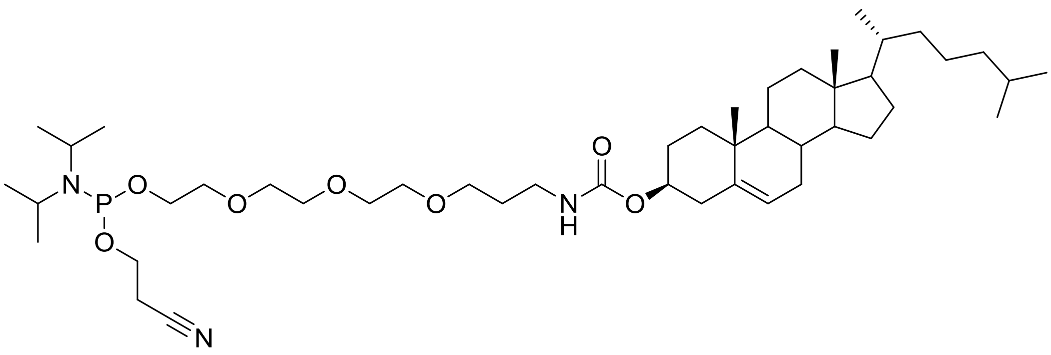

5'-Cholesteryl-TEG Phosphoramidite

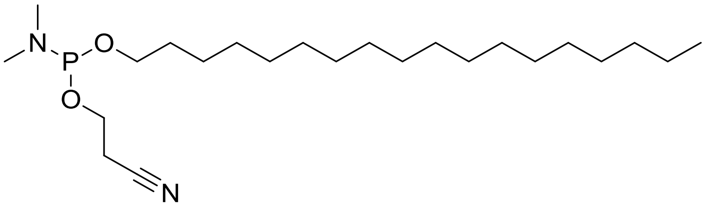

Single Chain C18 lipid phosphoramidite

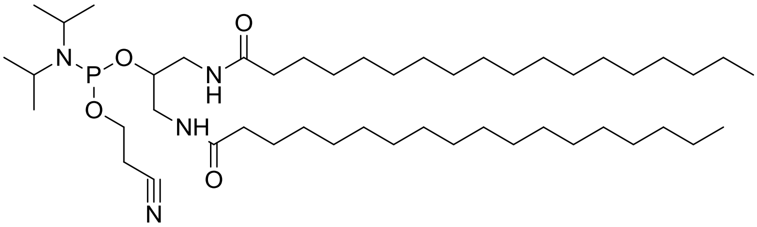

Diacyllipid phosphoramidite

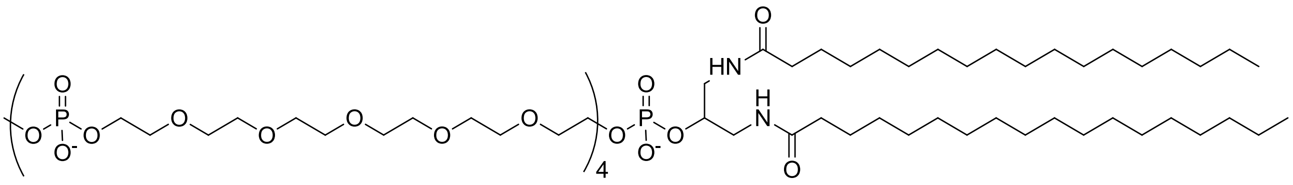

PEG-diacyllipid

Figure S1. Structures of lipophilic phosphoramidites and PEG-diacyllipid moiety used for ODN insertion analysis.

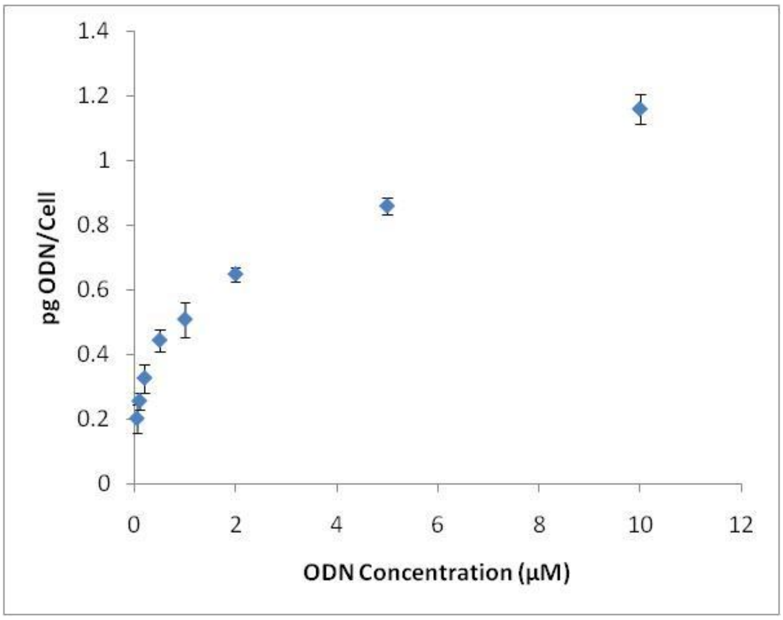

Figure S2. Cell surface ODN (pg) plotted as a function of ODN concentration.

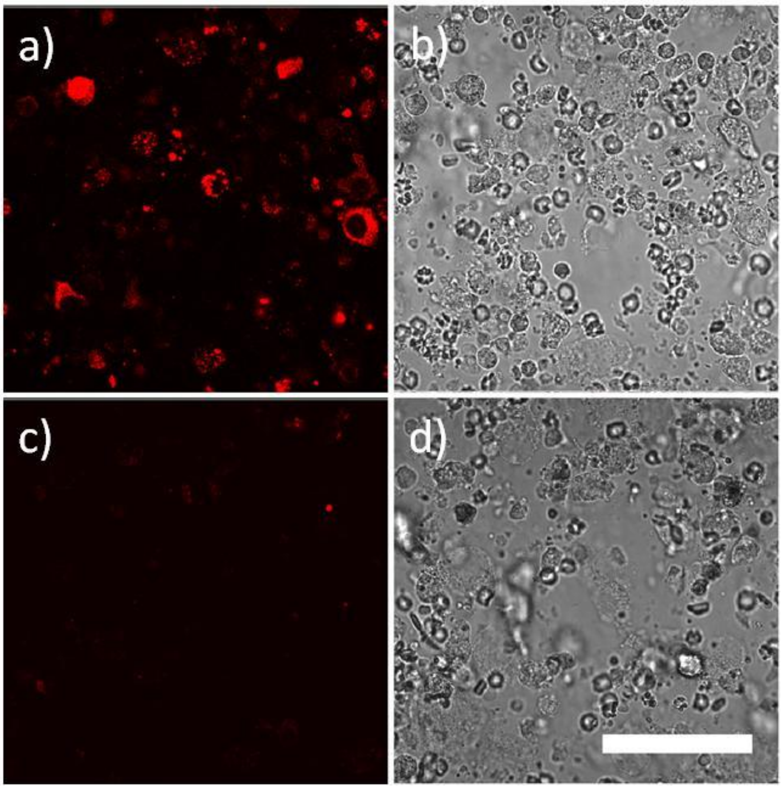

Figure S3. Confocal laser scanning microscope images of B16F10 tumor cells following intratumoral injections of Lipo-ODN-TMR (a, b) and Free-ODN-TMR (c, d). (a) and (c) Fluorescence images; (b) and (d) brightfield images. Scale bar: 50 µm.

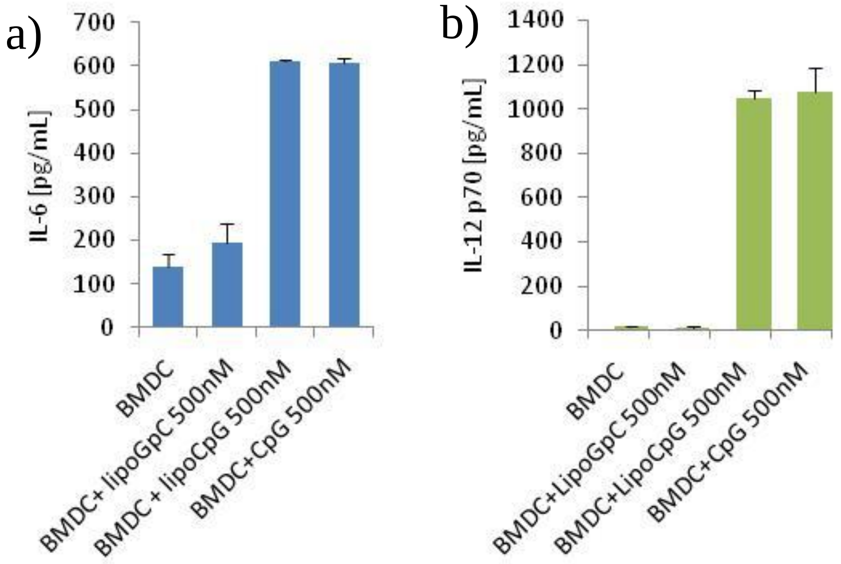

Figure S4. Relative immunostimulatory properties of various oligonucleotides used in the study. BMDC (day 9) were cultured for 12 h in the presence of various oligonucleotides (500 nM of each) as indicated. The secretion of proinflammatory mediators including IL-6 (a) and IL-12 (b) into culture media was assessed using ELISA.

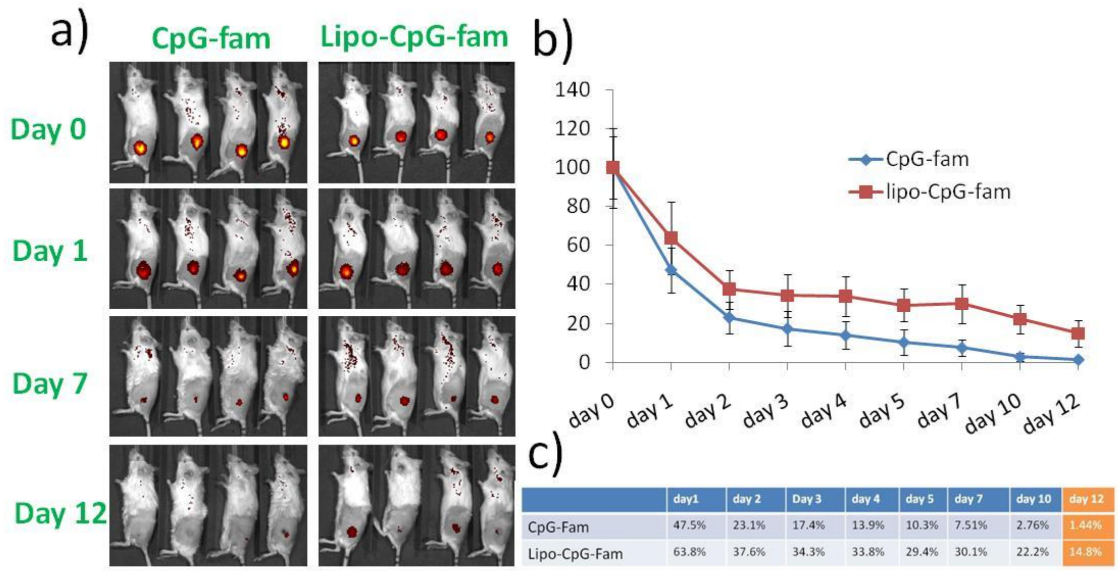

	day1	day 2	Day 3	day 4	day 5	day 7	day 10	day 12
CpG-Fam	47.5%	23.1%	17.4%	13.9%	10.3%	7.51%	2.76%	1.44%
Lipo-CpG-Fam	63.8%	37.6%	34.3%	33.8%	29.4%	30.1%	22.2%	14.8%

Figure S5. *In vivo* fluorescence kinetics after a single dose (20 μg) intratumoral injection of various dye labeled CpG derivatives. (a) IVIS whole animal imaging. (b) and (c) fluorescence quantifications in (a).
